# Delayed recognition of peripartum cardiomyopathy presenting with severe heart failure: a case report

**DOI:** 10.1093/ehjcr/ytaf306

**Published:** 2025-07-01

**Authors:** Nordini Asri, Suraya Abdul-Razak, Mohd Fazrul Mokhtar, Khairul Shafiq Ibrahim, Roqiah Fatmawati Abdul Kadir

**Affiliations:** Department of Primary Care Medicine, Faculty of Medicine, Universiti Teknologi MARA (UiTM), Kampus Sungai Buloh, Sungai Buloh, Selangor 47000, Malaysia; Department of Primary Care Medicine, Faculty of Medicine, Universiti Teknologi MARA (UiTM), Kampus Sungai Buloh, Sungai Buloh, Selangor 47000, Malaysia; Cardiovascular Advancement and Research Excellence (CARE) Institute, UiTM, Kampus Sungai Buloh, Sungai Buloh, Selangor 47000, Malaysia; Department of Emergency Medicine, Faculty of Medicine, Universiti Teknologi MARA (UiTM), Kampus Sungai Buloh, Sungai Buloh, Selangor 47000, Malaysia; Department of Cardiology, Faculty of Medicine, Universiti Teknologi MARA (UiTM), Kampus Sungai Buloh, Sungai Buloh, Selangor 47000, Malaysia; Department of Radiology, Faculty of Medicine, Universiti Teknologi MARA (UiTM), Kampus Sungai Buloh, Sungai Buloh, Selangor 47000, Malaysia

**Keywords:** Heart failure, Peripartum cardiomyopathy, Sacubitril/valsartan, Sodium–glucose cotransporter-2 inhibitor, Primary care medicine, Angiotensin receptor-neprilysin inhibitor, Case report

## Abstract

**Background:**

Peripartum cardiomyopathy (PPCM) is a rare but potentially fatal cause of heart failure that occurs towards the end of pregnancy or within the first 5 months postpartum, in the absence of other identifiable cause of cardiac dysfunction. It is characterized by left ventricular systolic impairment, with an ejection fraction (LVEF) typically ≤ 45%. While most cases are diagnosed shortly after delivery, delayed presentations can occur, leading to significant diagnostic challenges and complicate treatment.

**Case summary:**

We report a case of a 40-year-old multiparous (Para 3) woman, who developed progressive dyspnoea beginning 2 months after delivery. Despite two earlier medical care visits, her symptoms were initially misdiagnosed. She represented with overt symptoms of heart failure 1 month later. Investigations encompassing transthoracic echocardiography, cardiac magnetic resonance imaging, coronary angiography, and NT-proBNP confirmed PPCM with a severely reduced LVEF of 16%. She was promptly initiated on guideline-directed medical therapy (GDMT) for heart failure with reduced ejection fraction (HFrEF), including an angiotensin receptor-neprilysin inhibitor and a sodium–glucose cotransporter-2 inhibitor. At 3-month follow-up, her symptoms had resolved, and LVEF recovered to 56%.

**Discussion:**

This case highlights the diagnostic challenge of delayed-onset PPCM and reinforces the importance of maintaining high suspicion in postpartum women presenting with persistent dyspnoea. Early initiation of GDMT for HFrEF can lead to full functional recovery. Routine postpartum surveillance should include careful assessment for cardiopulmonary symptoms, even in the absence of overt risk factors, to prevent delays in diagnosis and management.

Learning points

- The presentation of dyspnoea during the peripartum period requires careful evaluation and appropriate follow-up, as delayed recognition may lead to missed diagnoses of serious conditions such as peripartum cardiomyopathy (PPCM).
- While sacubitril/valsartan and sodium–glucose cotransporter-2 inhibitors have demonstrated clinical benefits in heart failure with reduced ejection fraction (HFrEF), their use in PPCM is extrapolated from general HFrEF guidelines, and PPCM-specific evidence remains limited.

## Introduction

Peripartum cardiomyopathy (PPCM) is a rare, potentially life-threatening form of heart failure occurring in the last month of pregnancy or within 5 months postpartum, marked by new-onset systolic dysfunction (left ventricular ejection fraction LVEF ≤ 45%) without an identifiable cause.^1^ The incidence ranges from 1 in 15 000 to 1 in 100 live births, influenced by genetic, socioeconomic, and healthcare factors.^2^

Symptoms including dyspnoea, fatigue, and oedema often mimic normal peripartum physiology, contributing to diagnostic delays. Early recognition and treatment improve outcomes through neurohormonal modulation and ventricular remodelling.^3^

Management generally follows guideline-directed medical therapy (GDMT) for heart failure with reduced ejection fraction (HFrEF), including beta-blockers, angiotensin-converting enzyme inhibitors or angiotensin receptor-neprilysin inhibitors, and sodium–glucose cotransporter-2 (SGLT2) inhibitors. Their use in PPCM is extrapolated from HFrEF trials and remains off-label. This case illustrates a delayed diagnosis and subsequent recovery following GDMT, highlighting the need for clinical vigilance.

## Summary figure

**Figure:**
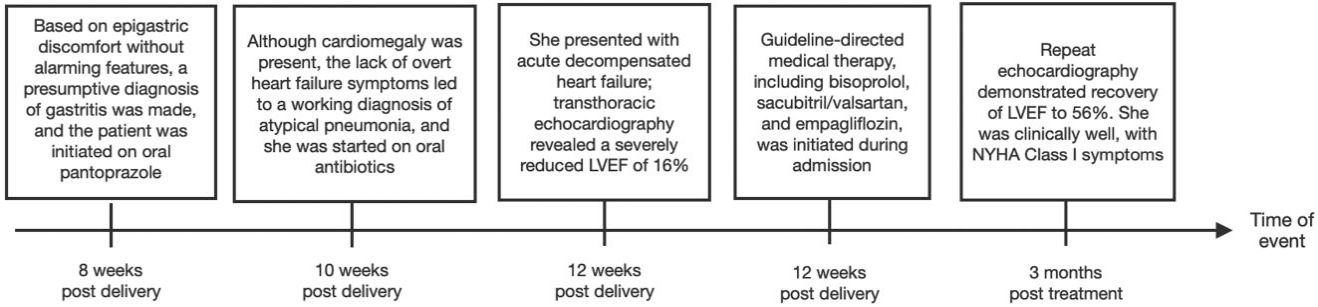


## Case presentation

A 40-year-old woman, para 3, presented to a primary care clinic with exertional dyspnoea starting 2 months postpartum. Initial symptoms of mild breathlessness and epigastric discomfort were managed as gastritis. Subsequently, she developed a productive cough without fever and was treated empirically for atypical pneumonia.

At 3 months postpartum, she represented with worsening dyspnoea, reduced effort tolerance, orthopnoea, paroxysmal nocturnal dyspnoea, abdominal discomfort, fatigue, and mild pedal oedema. She had no history of hypertension, diabetes, cardiac disease, or substance use. There was no family history of cardiomyopathy or sudden cardiac death. Her antenatal course was uneventful, with consistently normal blood pressure readings (114–126/68–80 mmHg), and no signs of pre-eclampsia. She was breastfeeding and not on regular medication.

She was mildly tachypnoeic (RR 22), SpO₂ 95% room air, blood pressure 144/95 mmHg, heart rate 107 bpm, without jugular venous distention. Capillary glucose was 9.7 mmol/L. Auscultation revealed dual heart sounds without any murmurs. Bibasilar crepitations and mild bilateral oedema were present. Hepatomegaly was evident with a tender liver palpable 4 cm below the right costal margin. Peripheral perfusion was intact.

**Figure 1:**
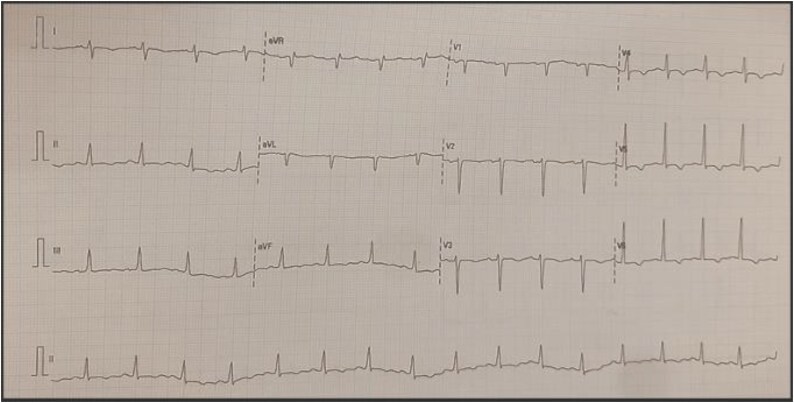
Electrocardiogram with widespread T-wave inversion. Reproduced with permission from Hospital Al-Sultan Abdullah, HASA UiTM, 2025.

**Figure 2:**
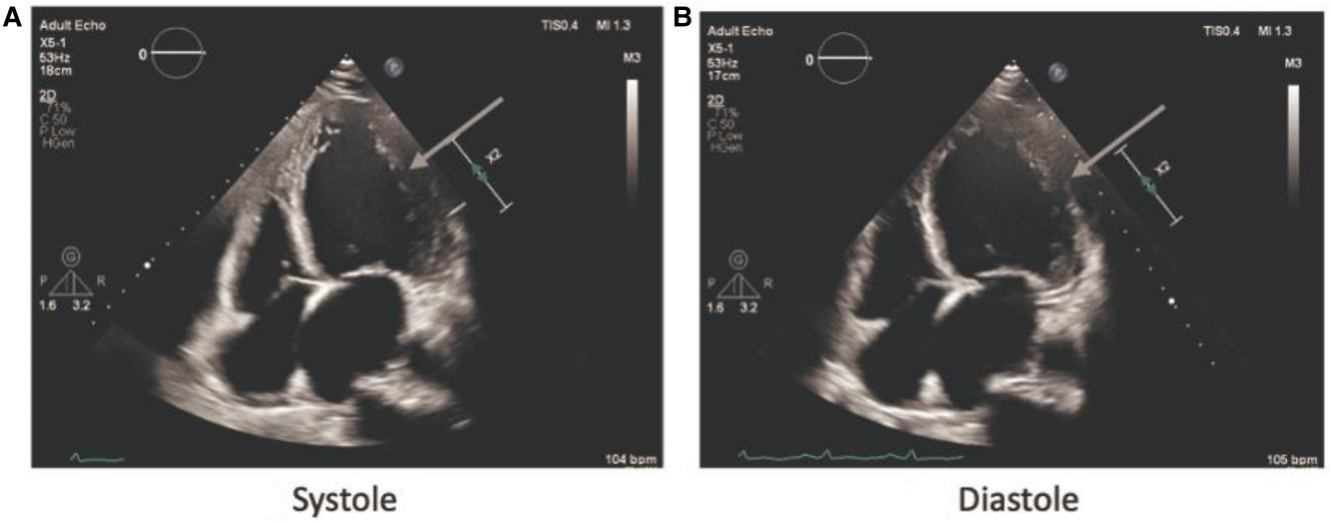
(*A*) and (*B*) showed evidence of left ventricle dilatation and global hypokinesia.

Electrocardiography (ECG) showed widespread T-wave inversions (see *Figure 1*). Chest x-ray revealed cardiomegaly and pulmonary congestion. Blood investigations showed normal haemoglobin, renal and liver profile, troponin T, thyroid function, and HbA1c (5.6%). NT proBNP was elevated at 4320 pg/mL. The echocardiogram showed a dilated left ventricle with an LVEF of 16%, global hypokinesia, pseudo-normal diastolic filling, enlarged left atrium, mild right ventricle dilatation, and moderate mitral and tricuspid regurgitation (see *Supplementary Video 1–5*). No pericardial effusion or regional wall motion abnormalities were noted (see *Figure 2*).

**Figure 3:**
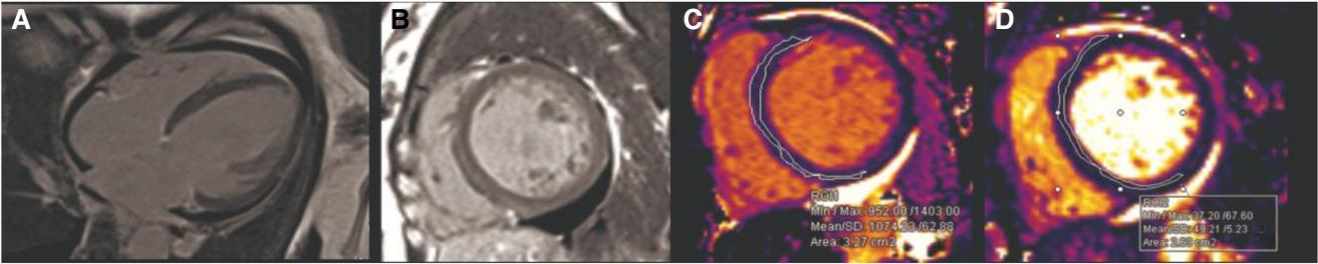
MRI of the heart showing (*A*) the longitudinal 4-chamber views demonstrate a dilated left ventricle with an end-diastolic volume of 126 mL/m². There is global hypokinesia observed on cine steady-state free precession images, with reduced left and right ventricular systolic functions of 20% and 22%, respectively. A pericardial effusion with a maximal thickness of 1.3 cm is also noted. (*B*) The late gadolinium short-axis image showed subtle intramyocardial enhancement, particularly in the septal and anterior walls. (*C*) and (*D*) Mid-ventricular septal measurements of T1 and T2 mapping demonstrate slight increases in T1 and T2 mapping values, respectively.

Coronary angiogram showed normal vessels. Cardiac MRI confirmed biventricular dysfunction (LVEF 20% and RVEF 22%). There was a subtle intramyocardial late gadolinium enhancement pattern consistent with dilated non-ischaemic cardiomyopathy. Mild diffuse oedema on T2 STIR images, along with a slight increase in native T1 and T2 mapping (see *Figure 3*).

She was admitted and started on intravenous furosemide and oxygen. GDMT was initiated with sacubitril/valsartan 24/26 mg twice daily, empagliflozin 10 mg once daily, bisoprolol 1.25 mg once daily, and spironolactone 25 mg once daily. These medications were gradually up titrated based on clinical response. The patient tolerated the treatment well without adverse effects. All medications were selected based on lactation safety. She responded well and she was discharged after 3 days.

At 3-month follow-up, she was asymptomatic (NYHA Class I) with normalized left ventricular function (LVEF of 56%). NT-proBNP was not repeated. She remained clinically stable on maintenance therapy.

## Discussion

This case highlights the diagnostic complexity of PPCM, where symptoms overlap with normal postpartum physiology. Initial misdiagnoses underscore the need for heightened suspicion in postpartum women with unexplained dyspnoea. Differential diagnoses include pre-existing cardiomyopathy, pulmonary embolism, and infection.^4^

The pathophysiology may involve inflammation, oxidative stress, angiogenic imbalance, and genetic susceptibility. Risk factors include advanced maternal age, multiple gestations, multiparity, and hypertensive disorders.^2,4,5^ In this case, advanced maternal age was the only apparent factor.

Echocardiography is the diagnostic cornerstone, complemented by NT-proBNP, ECG, chest imaging, and laboratory evaluation.^6–8^ Cardiac MRI aids in excluding other cardiomyopathies, although typical findings in PPCM are non-specific, such as global dysfunction, myocardial oedema, and subtle late gadolinium enhancement.^2,6^

Management follows HFrEF guideline,^9,10^ including diuretics, bisoprolol, spironolactone, SGLT2 inhibitor, and sacubitril/valsartan. Though effective in HFrEF, ARNI and SGLT2 inhibitors are off-label in PPCM and require cautious use.^2,11,12^ Counselling was provided to ensure continued breastfeeding and all prescribed medications were reviewed and supported by lactation safety data.^13^ The patient demonstrated marked improvement in LVEF from 16% to 56% and symptoms within 3 months.

Long-term follow-up is essential due to the risk of relapse, arrhythmias, or persistent cardiomyopathy.^2,14,15^ Recovery varies, with some patients normalizing cardiac function, while others experience lasting impairment or relapses.^2,14,15^ Counselling about future pregnancies is critical, as any subsequent pregnancy carries an elevated risk of recurrence.^2^ Our patient received a progestin-only implant (Implanon) to mitigate thromboembolic risks associated with oestrogen-containing methods.^2^ Breastfeeding was continued safely.

In summary, PPCM present diagnostic and management challenges. This case highlights the importance of postpartum vigilance and demonstrates favourable outcomes with timely GDMT. While HFrEF therapies show promise, further studies are needed to define their role in PPCM. Ongoing surveillance, cardiovascular risk reduction, and reproductive counselling are essential.

## Supplementary Material

ytaf306_Supplementary_Data

## Data Availability

The data underlying this article will be shared on reasonable request to the corresponding author.

## Lead author biography

Nordini Asri, born on 22 June 1990, is a Primary Care Medicine resident at Primary Care Medicine Department of Faculty of Medicine, UiTM, Malaysia.

## Supplementary material

**Consent:** The authors confirm that written consent for the submission and publication of this case report, including images, videos, and associated text, was obtained from the patient in accordance with COPE guidance.

**Funding:** None declared.
